# Health promoting sports federations: theoretical foundations and guidelines

**DOI:** 10.3389/fpubh.2023.1147899

**Published:** 2023-07-11

**Authors:** Aurélie Van Hoye, Susanna Geidne, Anne Vuillemin, Kieran Dowd, Iva Glibo, Sandra Heck, Bjarne Ibsen, Stacey Johnson, Melanie Kingsland, Sami Kokko, Aoife Lane, Linda Ooms, Marie Overbye, Catherine Woods, Geraldine Zeimers, Stephen Whiting, Mathieu Winand

**Affiliations:** ^1^Physical Activity for Health Research Cluster, Health Research Institute,Physical Education and Sports Sciences Department, University of Limeric, Limerick, Ireland; ^2^Faculty of Medicine and Health, School of Health Sciences, Örebro University, Örebro, Sweden; ^3^Université Côte d’Azur, LAHMESS, Nice, France; ^4^SHE Research Group, Technological University of the Shannon, Athlone, Ireland; ^5^European Sport NGO Youth, Stockholm, Sweden; ^6^Ecole Nationale de l’Education Physique et des Sports (ENEPS), Luxembourg City, Luxembourg; ^7^Southern Denmark University, Odense, Denmark; ^8^Department of Human and Social Sciences, Institut de Cancérologie de l’Ouest René Gauducheau, Saint-Herblain, Angers, France; ^9^University of Newcastle, Newcastle, NSW, Australia; ^10^Faculty of Sports and Health Sciences, University of Jyväskylä, Jyväskylä, Finland; ^11^Mulier Institute, Utrecht, Netherlands; ^12^Faculty of Health Sciences and Sport, University of Stirling, Stirling, United Kingdom; ^13^Louvain Research Institute in Management and Organization (LouRIM), Mor Sciences Faculty, UCLouvain, Louvain-la-Neuve, Belgium; ^14^WHO European Office for the Prevention and Control of NCDs, Copenhagen, Denmark; ^15^LUNEX International University of Health, Exercise and Sports, Differdange, Luxembourg

**Keywords:** health promotion, national sports federations, sports clubs, settings-based approach, guidelines, tools, interventions

## Abstract

**Background:**

Researchers and policy-makers have highlighted that the potential for organized sports to promote health has been underexploited. Sports clubs have limited capacity to promote health due to their voluntary nature and have called for support from their national sports federations. The present article provides guidelines, based on the theoretical principles of health promoting sports clubs and an analysis of practical tools and proven strategies, to support national sports federations to invest in health promotion (HP).

**Methods:**

A qualitative iterative study was undertaken, based on five 2-h meetings of a group of 15 international researchers in HP in sports clubs. Notes and minutes from meetings, as well as shared outputs were analyzed based on the health promoting sports club framework.

**Results:**

Guidelines developed for national sports federations to promote health includes a definition of a health promoting sports federation (HPSF), a description of how the settings-based approach to HP adapts to national sports federations, as well as practical applications of health promoting sports club’s intervention strategies. The analysis of existing tools also demonstrated that most tools are centered on a single dimension of health (social, mental, physical, spiritual or community), and often on a specific health topic. Furthermore, they do not cover HP as a continuous long-lasting process, but are generally short-term programs. The HPSF clarifies theoretical concepts, their practical implementation via case studies and outlines intervention components and tools useful for sports federations in their implementation of HP.

**Conclusion:**

The guidelines developed in this study are intended to facilitate national sports federations to acknowledge/understand, reinforce/underpin and foster current and further investment in HP.

## Introduction

Organized sports has largely been recognized and used by policy makers for its positive role as a powerful tool for the expression of political messages (1), and for facilitating wide-spread participation in sports and physical activity, with 12% of European citizens practicing sports in this setting (European Union, 2022). Research has also demonstrated that sport is a major contributor to the health of a nation (2). Specifically, evidence supports the contribution of sports participation to achieving international physical activity recommendations (3). Physical activity is largely recognized as a major health determinant (4, 5) contributing to improved health outcomes preventing non-communicable disease and improving mental, social and physical health (6). Nevertheless, the White Paper on Sports (European Commission, 2007) and the Global Physical Activity Action Plan 2018–2030 (World Health Organization, 2018) have both underlined sports clubs’ underexploited potential to promote health. Sports clubs have been defined as “private, non-profit organizations formally independent of the public sector, including volunteer members and a democratic structure, having sports provisions as their main aim” (7). Sports clubs could go beyond facilitating physical activity, by becoming health promoting settings (8–10) and by considering their potential to foster social, mental, physical and community health (11) However, evidence shows that this will not happen without support (12). The path to move from passive sports clubs providing physical activity opportunities to active health promoting sports clubs (HPSC) is long (13), represented as five stages in the settings-based approach to health promotion (HP) (14). Progressing through these stages requires formalized and systematic efforts, strategic focus and related marketing tactics to implement HP programs in collaboration with health actors (15). Indeed, the HPSC model calls for action on organizational (orientation, guidelines, policies and their implementation), economic (human and financial resources), social (vision, values and social norms in the club) and environmental (built environment and material) determinants of health at seven levels (from individual to policy makers) (16). In this regard, sports club managers expressed the need to have guidelines and support from their national sports federation (NSF) to implement HP (17–19), as programs promoting health through sports tend to be more complex than traditional sports development programs. This complexity is due to a need to deal with fundamental economic, cultural and health issues rather than a sole reliance on sports provision to achieve health impacts and outcomes (1). Sports clubs search for answers on appropriate methods to integrate multiple strategies on social, organizational, economic and environmental determinants across multiple health domains (social, mental, physical, spiritual, community) (20). In this regard, the application of the settings-based approach does provide a proper way to answer, even if HPSC implementation also face unintended health effects or may threaten the integrity of the organization (21), due to this complexity. For example, previous work has documented how sports clubs were confronted with a paradox in terms of feasibly implementing the safety policy, where stakeholders stated this policy was essential to ensure safe practice, but the cost to the club of its adoption would affect their ability to survive (22).

To date, research in this area has primarily centered on developing the HPSC model (16), and investigating how club managers and coaches promote health (23, 24). Research on Australian interventions among community sports clubs have shown effectiveness on behavioral outcomes (25), as well as cost-effectiveness (26, 27). Nevertheless, scant research has targeted NSFs (28, 29), and focused principally on programs dedicated to health topics, such as, safeguarding children (30), doping prevention (31, 32) and injury prevention (33). The presence, albeit limited, and the narrow awareness of HP as a global concept in international or NSF has been demonstrated (34), and its alignment to sports federation business is crucial for further investment (28, 35). Such findings suggest a need to improve political lobbying, project and change management capacity within sports federations to develop HPSF (34).

To determine the theoretical and empirical gaps in HP in NSFs, the present article focuses on the creation of the Health Promoting Sports Federation (HPSF) Guidelines, to answer the research question: How to evaluate and foster health promotion implementation among NSFs? This article serves as a basis for the guidelines, by (1) offering a theoretical conceptualization of a HPSF, (2) providing evaluation indicators for a HPSF, (3) developing practical applications of the intervention components from the HPSC framework for NSFs, and (4) reviewing how existing tools to promote health are linked to HPSF.

## Method

### Design

A qualitative, iterative design (36) was used to develop the HPSF guidelines, based on five steps: (1) defining a HPSF and applying the stages of the settings-based approach to HP a to NSFs, (2) creating HPSF evaluation criteria, (3) analyzing HPSF intervention components, (4) synthesizing tools supporting HPSF and (5) establishing HPSF guidelines.

### Participants

This work was led by an international research group, also acknowledged as authors in the present article, under the ‘Sports Clubs for Health’ working group of the health-enhancing physical activity network of the World Health Organization Regional Office for Europe. A project team, composed of the three first authors and the last author invited 20 researchers worldwide, renowned for their contribution to HP in sports, to take part in five 2-h virtual meetings (February 16th, March 26th, April 26th, May 24th, June 21st). Participants gave their informed consent to take part in the study and were informed about the ethical implications of participating in the project, through an email in January 2022. Each participant engaged in at least three meetings, with a mean participation rate of 12 (min = 9 and max = 14) per meeting.

### Data collection

The data was collected via feedback/notes and collaborative tools filled in by the first author during meetings, as well as by interactions and feedback through emails and an internal sharing platform between meetings. Agenda and minutes of each meeting were sent for approval, products generated from the meetings were shared for review and input from each participant was requested.

#### Step 1: defining HPSF and describing the stages of the settings-based approach to HP applied to NSFs

To develop the definition of a HPSF, the research group established an initial definition of a NSF that delineates its interconnections with other sport organizations in the pyramid sport setting, such as international sport federations (umbrella organizations for NSFs worldwide) and sport clubs, as well as the different organizational structures (sub-national level and local level). Then, using the definition of the settings-based approach to HP and of a HPSC (37) as a basis, the HPSF definition was derived. Furthermore, the different stages of the settings-based approach (14, 16) and the various descriptions of the relationship between sports and HP (38), which have previously been applied to sports clubs, were redefined in relation to NSF.

#### Step 2: creation of HPSF evaluation indicators

Evaluation indicators were identified based on two activities; an initial brainstorming session on criteria that could help to inform how HP was developed in sports federations, before a working meeting to classify these indicators based on the four already defined determinants of health from the HPSC model (16): (1) organizational determinants (NSFs guidelines about HP including policies, rules and regulations are provided to affiliated sports clubs), (2) social determinants (NSFs vision, values and philosophy are in relation to those of society), (3) environmental determinants (NSF offers support for safe, supportive and sustainable infrastructure, green spaces and playing fields for affiliated sports clubs), and (4) economic determinants (NSF provides financial and human resources for HP to affiliated sports clubs).

#### Step 3: development of HPSF intervention components

The intervention components (i.e., actions to be undertaken by NSF to develop HP) from the HPSC framework (16), that were previously classified under the responsibility of NSFs, were selected to ground the present work in an existing theoretical model, the HPSF framework (16). The research group first created a template of information needed for NSFs to implement each component, and determined the following: (1) how they were linked with NSFs’ previous experiences and actions, (2) what was the purpose of their implementation, (3) how they should be implemented according to the HPSF stages and (4) what role each stakeholder in the HPSC framework plays in its implementation. The template was discussed during two meetings, and then completed by the first author, with each component review by a member of the research group who had the highest level of expertise.

#### Step 4: review of tools supporting HPSF

To select tools supporting HPSF, a systematic search was conducted on the Erasmus+ website,^1^ using the keywords “health promotion” and “sports.” Inclusion criteria were: (1) to target HP or health topics in organized sports, (2) to include an English version, (3) to propose a tool (training, game, booklet, etc.) as an output, (4) to cover more than a single sport and (5) to constitute a practical document, not only policy recommendations. Exclusion criteria were (1) not targeting organized sports but physical activity in general, (2) no final tool availability in English language. An excel spreadsheet was developed for data extraction and circulated among the participants, to add details extracted from known tools based on the given inclusion criteria. When selected, the tool was fully reviewed by research group members, including website presentation, tool presentation and content. The review process for the tools was defined during two meetings. Meeting 2 focused on defining the templates for analysis using four categories: (1) description of the tool (weblink, language, link to HP, topic covered, date of release), (2) pedagogy of the tool (objectives, content, HPSF stage reached using the tool, person using and person targeted by the tool, type of tool, time estimated to complete and to implement, need for a trained person), (3) production and evidence (tool creation process, quality of evidence), (4) link with HP approach and theoretical framework (strategies of the Ottawa Charter and of the HPSC framework mobilized by the tool). Meeting 4 focused on discussing inclusion criteria for some tools where doubts had been expressed and adapting the template based on a review of the two tools. A double peer review process was undertaken on the chosen tools; a first draft analysis was produced for all tools by the first author and reviewed by a member of the research group.

#### Step 5: finalization of the HPSF guidelines

All of the different work sections were compiled into a single document. The design was reviewed by research group members, as well as external sports and health experts, including three French sports ministry members, two project managers from the French Public Health agency and three representatives from NSFs (France, Sweden, Luxembourg). The use and dissemination strategy were reviewed by the research group members during their last meeting.

### Data analysis

Notes and productions were analyzed by the first author using a deductive approach, based on the HPSC framework as a theoretical basis (16). The data analysis was conducted between each meeting, based on participants’ answers, as well as on the minutes from meetings sent to the research team. The results and output from each step were sent again to the research team/participants. They could provide their feedback before or at the beginning of the next meeting. After the five meetings, all notes were collated and reviewed twice by the first author (to become familiar with the content and to verify comments to include in the final guidelines). The research team read the final product and provided feedback twice. This feedback has been considered in the final guidelines. Data validity was addressed by having all participating researchers validate the final version of the guidelines (ensuring triangulation and respondent validation). In addition, a cross-country comparison of the guideline’s applicability was undertaken based on participants’ experience (triangulation of context) and an iterative constructive process of including existing literature on HPSC at each level, as well as during the case study search. Finally, data reliability has been facilitated by ensuring appropriate wording was used in the guidelines, and through collective validation of the final version of this article.

## Results

### Step 1: definition of a HPSF and of the settings-based approach stages applied to NSFs

**Figure 1:**
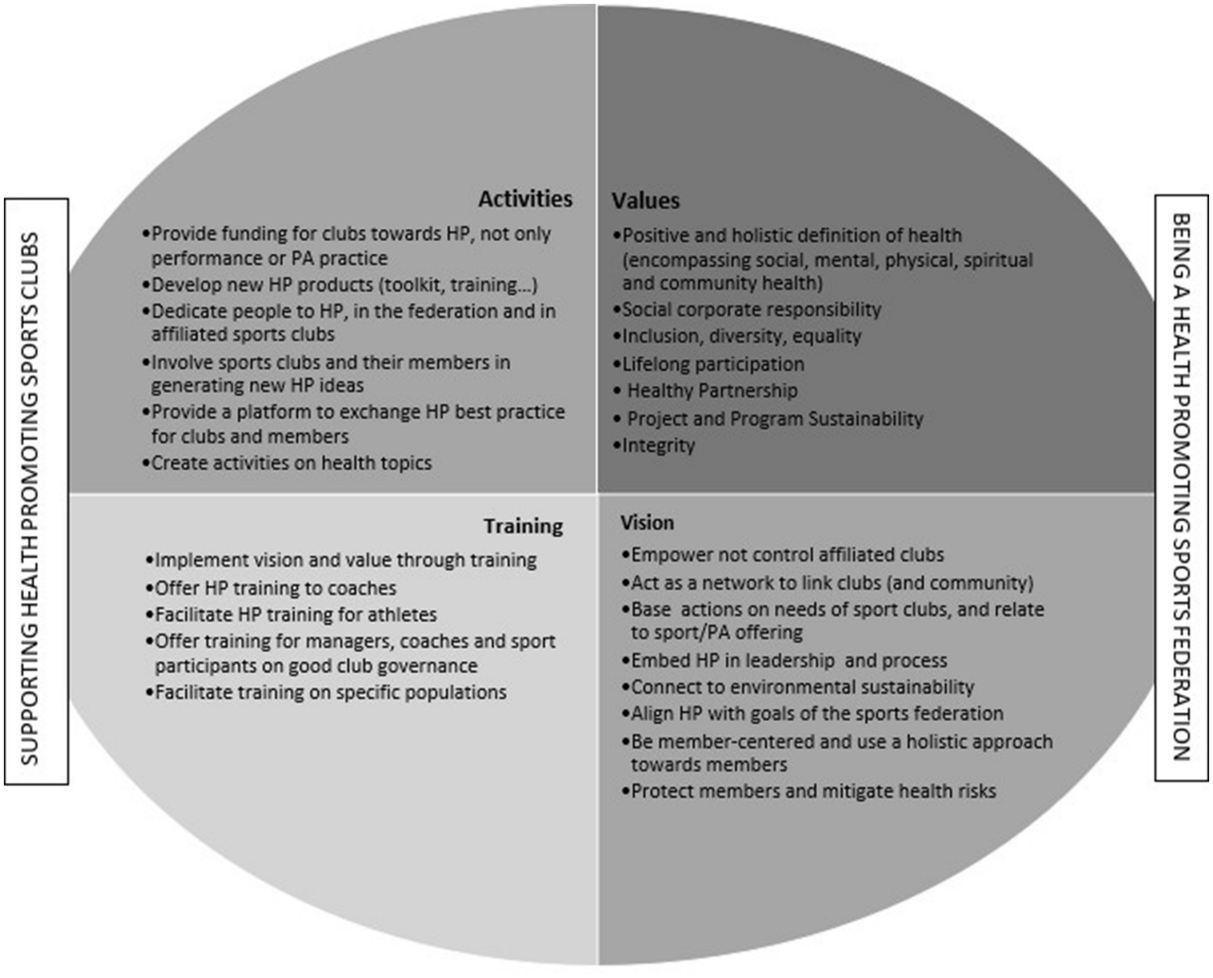
Description of a HPSF.

The research group adopted the Institut National de la Jeunesse et de l’Education Populaire (INJEP; National Insitute for Youth and Popular Education) definition of sports federations: “National sports federations are responsible for planning and managing their sports at a national level, through an organization based on membership of affiliated clubs. A sports federation organizes and promotes the practice of it(s) discipline(s), from leisure activities to high level sports.”^2^ This definition was considered as the most inclusive of all types of NSF, including non-Olympic ones, as well as including different types of sports practice. After brainstorming the different activities and processes in place in NSFs, a HPSF was defined by the research group as a “national sports federation that considers health in its values, vision and leadership, as well as in its activities and training” (see Figure 1 for details). There are two primary aspects of a NSFs focus towards becoming health promoting: “to be a health promoting sports federation” (considering health in all policies, decision making processes, structures and activities) and “to support their affiliated clubs to become health promoting” (invest in programs, guidelines, toolkits, human resources helping affiliated club to promote health). Moreover, the application of the five stages of the settings-based approach to health promotion (14) can offer insight to NSFs on how to progress towards becoming a HPSF (see Table 1 for details).

**Table 1:** Stages of health promoting sports federations inspired by (14).

	Stage	Core perspective	Definition	Action of national sports federation
0	Null			No communication on legal requirements related to health/reducing health risks.
1	Passive stage	The problem and solution rest within the behavior and actions of individuals	Safe sports offer fostered by sports federations	Promoting safe and secure sports activities (legal requirements to protect sports participants: doping, warming up, etc.). Sports clubs may independently undertake initiatives in HP.
2	Active stage	The problem lies within the behavior of individuals, some solutions lie in the setting	Sports federations promoting health	Promoting health benefits of sports for sports clubs and participants and signposting HP activities to sports clubs, but not actively engaging in HP programs.
3	Transmissive stage	The problem lies within the setting, the solutions lie in learning from individually-based projects	Health Promotion in Sports Federations	Sporadic HP events organized by the sports federation (event day), on a single health behavior or topic; Encouragement from sports federation to sports clubs to deliver club-based HP activities.
4	Organic stage	The problem lies within the setting, the solutions lie in the actions of individuals	Sports Federation Health Promotion programs	Dedicated programs developed by the sports federation to enhance sports club’s activities around HP. The sports federation is actively involved in becoming a platform for sports clubs and external partners to meet for HP
5	Comprehensive approach stage	The problem and solutions lie in the setting	Health Promoting Sports Federation	HP is integrated in the vision, values, activities and training courses of the sports federation. Health is considered in the organizational structure and decision-making processes at all levels of the sports federation. The sports federation invests ongoing resources in HP in the long-term, to promote health beyond health topics. The sports federation collaborates with external partners on HP. The sports federation’s policies are based on monitoring and evaluating previous activities.

### Step 2: creation of HPSF evaluation indicators

The research group created nine categories of HPSF evaluation indicators through brainstorming (engagement from NSF, HP policy implementation, dedicated training on HP, dedicated HP programs, presence of safeguarding for members, presence of HP or prevention for (elite) athletes, financial investment, visibility, governance structure) and emphasized the need to ground them in a theoretical model (Ottawa Charter, Global Physical Activity Action Plan, HPSC model). For each category, work was undertaken to clarify the indicators, the questions used to evaluate them and the data sources an NSF could use. A final list of 18 indicators based on the four HPSC health determinants (nine organizational determinants, three environmental determinants, three economic determinants, and three social determinants; see Table 2 for details) were integrated into the HPSC model, which was approved during the second meeting. These indicators have been designed to work on the whole system of the NSF, impacting both policy framing and practice, as well as the decision-making process, as described in the settings-based approach to HP. Moreover, these indicators served as a basis for the case study analysis in chapter 2 of the guidelines (on case studies), and can be used as a self-evaluation grid to choose the strategies presented in chapter 3 (on implementation strategies) and the tools presented in chapter 4.

**Table 2:** Indicators of a health promoting sports federation.

Indicators	Questions	Data sources
*Organizational determinants*		
Engagement from national sports federation (importance)	How important is HP for the national sports federation?	Policy document Board discourse Code of conduct
HP policy planning	What types of HP activities does the national sports federation commit to in the policy documents? How many HP activities does the national sports federation plan per year?	Policy document
HP policy implementation	How many HP activities does the national sports federation undertake per year, based on policy? How many sports clubs and their members take part in the activities? What are the characteristics of these clubs and individual members?	Policy document Annual Report
HP monitoring and evaluation	Are HP activities monitored? Are their outcomes and impacts evaluated? What are the HP perceptions of the sports clubs and members taking part in them?	Annual report, strategic monitoring and evaluation plan
HP programs	Does the national sports federation have dedicated programs related to health topics or HP? How are they implemented?	Website
Training on HP	How is HP embedded in mandatory coach training? Is there any specific training related to health topics or HP to clubs/club members??	Website, training course documents
HP decision making process	Who is appointed within the national sports federation for HP? Who is involved in the decision-making process about HP activities and how are decisions made?	Website, policy document, notes of (board) meetings
Participative approach	Can the sports clubs or club members be involved in the sports federation’s decision-making process?	Description of the organization or human resources structure
Presence of safeguarding for members	Are there opportunities for sports clubs members to report misconduct to the national sports federation?	Code of conduct, critical incident plan
*Environmental determinants*		
Presence of HP or prevention measures for (elite) sports participants	Are specific HP regulations in place for training and/or competitions?	Website; code of conduct
Presence of a first aid kit, defibrillator	Are affiliated clubs equipped with a first aid kit and a defibrillator?	Annual report, survey, program description
Presence of guidelines for safe and sustainable environment	Are affiliated clubs encouraged to invest in safe and sustainable infrastructures and material?	Annual report, survey, program description
*Economic determinants*		
Financial investment (human and material)	How much does the national sports federation invest in HP? Is HP a priority in terms of resources?	Budget, annual report, human resources
Presence of corporate social responsibility investment	Does the national sports federation have a corporate social responsibility plan or activities?	Strategic plan, annual report
Presence of partnership with organization supporting HP	Does the national sports federation partner with HP organizations or HP professionals? Or with other organizations to invest in HP?	Strategic plan; programs description; website
*Social determinants*		
HP values	Does the national sports federation promote HP values (see figure above)?	Website, policy document, program description
Communication of HP	Is HP part of internal (within the national sports federation and affiliated clubs) and external (public and partners) communication strategies?	Mention of HP or health topics in newsletter, website, press release, on social media
Visibility of HP	Is HP explicitly mentioned in the communication and policy document of the national sports federation?	Social media, website

### Step 3: development of HPSF intervention components

Intervention components were operationalized to support practice-based use. The final full description of each of the 26 intervention components can be found in the guidelines (Table 3 summarizes how each intervention component can be implemented by NSFs at each HPSF stage). The decision was made to retain the original formulation of the intervention components, as well as not to create a new one, to keep consistent with the HPSC framework and to develop a practical application of an existing theoretical model, rather than the creation of a new theory (Table 3). This work has raised the issue of describing actions that could be seen as an example to implement any health behavior and emphasize the progression from an individual change driver in a passive organization to an active organization fostering individual health and well-being. This question has been answered on choosing to either use the formulation mentioning a single health topic, which is limiting in regard to applying a holistic HP approach, or in choosing to use the umbrella concept of HP as a keyword for investment in multiple behaviors and health topics. Furthermore, as the intervention components belong to a strategy, we found the intervention components to be redundant, which was considered as acceptable, as NSFs will have to choose which to implement and will probably not target all of them at the same time.

**Table 3:** Application of the HPSF stages to HPSC intervention components (16).

Name of intervention components	Stage 1: safe sports offer fostered by sports federations	Stage 2: sports federations promoting health	Stage 3: HP in Sports Federations	Stage 4: sports federation HP programs	Stage 5: health promoting sports federation
COM3: Ensure internal club communication on HP	Inform sports clubs about legal requirements and let them communicate	Propose specific signage or message to communicate on HP within sports clubs	Organize specific event to disseminate on health topics and encourage clubs to signpost message on HP topics	Propose guidelines on how to create a communication plan on HP and communicate regularly on clubs’ HP activities	Disseminate exemplar practices on sports federation’s website, a specific hashtag in sports federation’s communication, and a platform to share experiences and collect inputs from affiliated sports clubs
COM4: Ensure the club communicates with the external community on HP	Inform the community about legal requirements while entering sports facilities	Propose specific signage or message to communicate on HP	Offer a showcase of sports clubs’ HP projects in the sports federation	Propose guidelines to support sports clubs to develop their external communication strategy	Disseminate exemplar practice on sports federation’s website, a specific hashtag in sports federation’s communication, and a platform and strategy to share information
EDU1: Support the managers and coaches to actively engage in gaining knowledge and skills to promote health	Remind the regulations of coaching certification to managers and coaches	Provide a list of readings and training enhancing coaches and managers HP skills and knowledge	Encourage coaches and managers to take part to training in HP, as well as propose list of reading, online resources and platform of exchange	Propose training in regard to delivering specific HP programs, to ensure a quality of delivery	Accredit public health organization training and support in coaches and managers training certification process and provide funding incentive
EDU2: Tailor the support to the managers and coaches individually in relation to the sports participants they coach (mentoring, courses, online tools)	Transfer legal requirements to coaches	Provide information (e.g., leaflets) on how coaches or managers can enhance their knowledge in managing specific health topics	Organize events or conferences on promoting specific health topics	Promote specific programs for different health topics, including different education options (e.g., online tools, leaflet, games)	Include specific programs in sports federation policy. Offer a shared experiences platform, online course.
EDU3: Encourage the managers and coaches to support each other to promote health	No action	Provide information on sports clubs’ consultation process and advantage	Organize events in sports clubs and at local level to support collective dynamics on HP	Offer support and programs to help the development of sports clubs’ participative approach and experience sharing	Request from sports clubs to have a HP commission and a representative at executive board. Have a mentoring system for coaches and managers
EDU4: Propose a variety of ways for the sports clubs to raise awareness about HP	Propose signage and posts on legal requirements for sports practice	Propose ready to use signage, posts on social network and press releases on HP	Propose events to raise awareness on HP	Propose a recognition for HP programs, as well as program promotion materials	Propose a labeling system on HP, including events, signage and community of practice
EDU5: Create tools and training courses to support HP in sports clubs	Propose training on knowledge about legal requirement for sports delivery	Provide specific training on health topics (managers, coaches, volunteers)	Provide lifelong training on HP (managers, coaches, volunteers)	Propose specific training for HP program delivery	Integrate HP training into all mandatory sports federation training, as well as a specific certification system (managers, coaches, volunteers)
EXP5: Rely on existing, evidence-based HP tools	No action	Communicate on existing evidence-based tools	Provide a database of evidence-based tool, guidance on how to implement them	Base HP program on evidence base practices and tools	Have partnership with academics to root evidence-based research into sports federation strategic plan and stay informed about the last produced evidence
EXP7: Rely on other clubs’ experiences when developing HP actions	No action	Provide example of good practice on sports federation website	Create an open working group and committee to support experience sharing (i.e., having an online platform)	Co-construct programs with sports clubs and pilot them before implementation, share piloting club’s experience	Have a label program, which value sports club’s investment and offer them to participate to a mentoring system of other clubs, as well as a library of these projects to be shared
GLS1: Define the goals of HP	Have goals based on the respect of legal requirements	Have goals on specific health topics (citizenship, injury prevention, doping) in the sports federation’s strategic plan	Guide sports clubs on how to define their specific goals for HP	Consider HP as a specific goal, which covers different health topics and programs under an umbrella concept	Consider HP as a transversal goal in the policy development and have goals of integrating health in every decision-making process
MOB1: Mobilize sports champions to support the development of HP within your club	No action	Communicate on HP actions from high level athletes or exemplar sports clubs	Recruit an appropriate ambassador for a specific health topic program or projects and associate its image with the program Educate and support the champion on the health topic	Involve the champion in HP program and its dissemination, including participations at events	Encourage and support each champion to develop specific HP actions or programs in their clubs, by engaging them in program design, implementation and dissemination
MOB5: Mobilize local decision-makers and elected officials to promote health within the sports club	No action	Communicate to the local decision-makers sports clubs’ activities in regard to HP	Organize events with local decision makers on HP	Set partnership with local municipality and policy-makers in terms of HP commitment and implementation	Through partnership, inscribe sports club’s HP activities in local decision-makers policies and actions, as well as being members of specific interest group at local level
MON5: Review the HP policies of the sports clubs	Ensure sports clubs policy entails legal requirements	Communicate on the importance of reviewing HP policies of the sports clubs	Encourage sports clubs to review their HP policies, and communicate on guidelines to do it	Put a report system in place, where sports clubs can upload their development plan, especially the HP part	Provide guidance on how to review HP policies, good examples, use a report and accreditation system to acknowledge sports club’s HP policies
MOT3: Take coaches’ motivation for coaching and their future expectations into account	No action	Communicate on coaches’ qualifications regulations and training requirements	Encourage clubs to find a balance on coaches’ appointment to the group they will coach, taking their expectations into account	Provide guidance on how to retain volunteer coaches, establish good working conditions for coaches, as well as on training	Establish template for coaches’ career plan and mentoring system for coaches
MOT4: Strengthen coaches’ autonomy to promote health	Inform coaches about legal requirements	Communicate on HP policies and activities of the NSFs	Organize events underlining coaches role towards HP	Provide guidance, training and program to support coach’s investment in HP	Generate peer learning, working group and platform with resources for coaches to promote health
PAP1: Identify and call attention to HP actions of individuals	No action	Communicate on HP activities to sports participants	Organize events to recognize individual HP good practices	Propose empowerment program for sports participants, and make them visible to sports clubs	Offer a system in place to report exemplar action and offer an accreditation for exceptional individual’s contributions towards HP
PAP3: Identify and call attention to management HP actions	No action	Communicate on health topics	Organize events to recognize club HP good practices	Ensure managers are able to recognize and value HP actions, and train them on empowerment	Offer a system in place to report exemplar programs and offer a label for exceptional contribution toward club’s HP
PART1: Identify partners for HP (clubs, agencies, regional authorities, health professionals)	No action	Communication on HP organization name and mission	Propose guidelines on how to develop partnerships for promoting health topics	Propose a national partnership with HP actors, for the development of specific programs	Include national partners to support a health in all policy development and implementation, and their representatives at local level to foster sports club’s HP development
PLAN6: Encourage sustainable HP actions	No action	Communicate on criteria for sustainable HP actions	Propose guidelines and support to apply sustainable HP criteria to program implementation	Ensure that each HP program entails a section on sustainability and its monitoring	Encourage clubs to provide input on the use of sustainable HP criteria for their actions, as well as strategies for scaling up their development plan. Have a dedicated section in sports federation policy and indicators to evaluate sustainability of policy evaluation
PLAN8: Plan future actions based on the evaluation of current actions	No action	Identify and communicate to sports clubs’ members on previous actions and programs, which could be used by other sports federations or clubs	Propose guidelines on action planning in regard to specific health topics	Propose a systematic evaluation and reporting of sports club’s HP actions, with success indicators for program implementation	Establish a monitoring system of sports club’s HP actions, with an open access data base, where sports federations, clubs can get inspired by previous actions, as well as guidance on how to scale up specific actions
RES3: Review current skills and knowledge available to promote health	No action	Communicate a list of programs and training targeting health topics or HP	Provide guidance and checklist on skills requested to organize HP events	Identify sports federation employee profile, skills and knowledge to invest in HP	Have a directory of sports federation employees, as well as volunteers in sports clubs that could support HP implementation
RES4: Identify and mobilize tools for HP development within sports clubs	No action	Communicate on tools that have been published to promote health in sports clubs	Propose guidance and tools to promote specific health topics	Provide a database of tools for HP, disseminate them and guide sports clubs to implement them	Evaluate the quality and transferability of tools, provide guidance on implementation, as well as training on how to use them
RES5: Identify the funding that can be used for HP actions	No action	Communicate on available funding stream from different organization	Guide sports clubs to identify funding on specific health topics	Provide a database of funding, deadline and guide sports clubs to draft funding application	Evaluate the relevance of funding, provide guidance on implementation, as well as training on how to apply and implement them
RES6: Establish a national resource site for HP within sports clubs	No action	Have a page dedicated to HP on sports federation website	Have a specific section on sports federation website, including case studies of good practice	Have a specific section on sports federation website, promoting national HP programs	Have a specific section on sports federation website, cases studies of good practice, tools, evidence and links to external websites, a forum for discussion
RES7: Establish a national spokesperson for HP within sports clubs	No action	Communicate the list of employees and function in the sports federation	Have different employees playing a role on specific health topic	Have a specific employee appointed for HP, linking people and programs to the HP concept	Have a board representative, as well as a specific commission being appointed for HP in the NSF
RES8: Create and host a regional and local network of HP mentors within sports clubs	No action	Sharing of sports club’s managers contact details, when they propose HP actions	Have different employees playing a role on specific health topic	Share a profile of the sports clubs’ managers who volunteer to support other sports clubs and offer an annual meeting	Have a meeting with mentors every three to six months, training provided on HP and evidence update, as well as link with national employees, to create a community of practice

### Step 4: analysis of tools supporting a HPSF

Among the 117,501 Erasmus+ projects identified between 2015 and 2022, 336 projects included both HP and sports as keywords. Sixty projects were collaborative partnerships (staff training and youth exchange projects were excluded because no tool was produced). Among the 60 included projects, 26 targeted sports organizations and provided links to outputs or tools, leading to their inclusion in this review. In addition to the 26, the members of the research group provided links to additional tools they had identified as relevant. Eliminating duplicates and based on a full-text review, 28 tools were retained and presented in the guidelines. Reasons for exclusion of projects included: they were not directed towards sports federations (*n* = 7), tools were inaccessible (*n* = 4), content was not practical enough (n = 1), was not focusing on HP (*n* = 5), focused on a single sport (*n* = 1).

Of the 28 tools, 16 were documents or leaflets, two were websites, one was a game, four were online trainings and five a combination of two or more types of tools (Table 4). Social health was considered in 13 tools, mental health in 12 tools, physical health in 8 and governance or capacity building in 5 tools, while 4 tools targeted more than one dimension of health action. Regarding the evidence used to build the tool, 18 were research based, 24 practice based and 5 built with stakeholders, whereas 3 tools included a combination of these. On average, tools targeted three of the five Ottawa Charter (39) strategies (*n* = 11 for building public health policies, n = 25 for creating supportive health environment, *n* = 18 for strengthening community action, *n* = 20 for developing personal skills and n = 8 for reorienting health services), where three covered all five strategies. On average, six strategies from the HPSC framework were developed in the identified tools (min = 1 and max = 13), with 16 based on planning, 17 on education, 18 on resources, 9 on feasibility, 11 on goals, 18 on mobilization, 8 on monitoring, 15 on participative approach, 17 on partners, 15 on communication, 11 on dynamic, and 8 on experience.

**Table 4:** Analysis of the tools in regard to HPSF.

Name of the tool	Health topic	Type of tool	Type of evidence	Ottawa charter strategies	Intervention strategies
Stepping in: a bystander action toolkit to support equality and respect at work	Social Health: Gender Equity	Leaflet and website	Practice based evidence	Create supportive environment Develop personal skills Re orient health services	Planning Education Resources Feasibility Goals Mobilization Monitoring Motivation Participative approach Partners Communication
EU guidelines on dual careers of athletes	Dual Career (elite athletes)	Leaflet	Research and practice-based evidence	Build healthy public policy Create a supportive environment for health Develop personal skills	Planning Education Resources Monitoring
GAA Healthy Club Manual	Health Promoting setting	Leaflet	Practice based evidence	Build healthy public policy Create a supportive environment for health Strengthen community action for health Develop personal skills Re-orient health services	Planning Education Resources Dynamic Experience Feasibility Goals Mobilization Monitoring Motivation Participative approach Partners Communication
Good governance game	Good governance	Game and online training	Research and practice-based evidence and using a participative building approach	Create supportive environment for health Develop personal skills	Dynamic Mobilization Participative approach Communication
Sports diplomacy course	Sports Diplomacy	Online training	Practice-based evidence	Create supportive environment for health Strengthen community action for health Develop personal skills	Planning Education Resources Feasibility Goals Mobilization Monitoring Motivation Participative Approach Partners Communication
International safeguards for children in sports	Child protection	Leaflet	Research and practice based	Build healthy public policy Create a supportive environment for health Strengthen community action for health	Planning Resources Monitoring Motivation Partners Communication
Mental health charter for physical activity and sports	Mental health	Leaflet	Participative building approach	Build healthy public policy Create a supportive environment for health Strengthen community action for health	Planning Education Resources Goals Mobilization Partners Communication
Mental well-being coaching toolkit	Mental well-being	Leaflet	Research based and practice based, using a participative building approach	Create supportive environment for health Strengthen community action for health Develop personal skills Re-orient health services	Education Resources Dynamic Experience Motivation Participative approach Partners Communication
Outsports tool	Inclusion and diversity in sports	Leaflet and game	Practice based evidence	Create supportive environment for health Develop personal skills	Planning Education Mobilization Motivation
Pro Safe Sports+ Training Kit	Prevention of sexual violence in sports	Leaflet, video clip, online resources center	Research and practice-based evidence	Create supportive environment for health Strengthen community action for health Develop personal skills	Planning Education Mobilization Motivation
Sports and sustainable development goals	Sustainable development	Leaflet	Research and practice based evidence, using a participative building approach	Build healthy public policy Create a supportive environment for health Strengthen community action for health	Planning Education Resources Dynamic Experience Mobilization Motivation Participative approach Partners
Sports for active citizenship toolkit	Participative approaches, active citizenship	Leaflet	Practice based evidence, using a participative building approach	Create supportive environment for health Strengthen community action for health Develop personal skills	Resources Dynamic Mobilization Participative approach
Sports for protection tool	Social inclusion, social cohesion	Leaflet	Research and practice based evidence, using a participative building approach	Create supportive environment for health Strengthen community action for health Develop personal skills	Planning Education Resources Dynamic Feasibility Goals Monitoring Motivation Participative approach Partners
Sports without Doping! A training tool for Anti-Doping Junior Ambassadors	Doping prevention	Leaflet	Research and practice-based evidence	Develop personal skills	Education
Staying in side: how to stop match-fixing	Match fixing	Leaflet	Practice based evidence	Create supportive environment for health	Resources Partners Communication
The good sports program	HP	Website	Research and practice-based evidence	Build healthy public policy Create a supportive environment for health Strengthen community action for health Develop personal skills	Planning Education Resources Dynamic Experience Feasibility Goals Mobilization Monitoring Motivation Participative approach Partners Communication
Community sports for children and youth planning toolkit	Sports and physical activity	Leaflet	Practice based evidence	Create a supportive environment for health Strengthen community action for health	Planning Resources Experience Feasibility Goals Monitoring
Everyone wins community sporting clubs	Gender equality, diversity and inclusion	Leaflet	Practice based evidence	Build healthy public policy Create a supportive environment for health Strengthen community action for health Develop personal skills Re-orient health services	Planning Education Resources Dynamic Experience Feasibility Goals Mobilization Monitoring Motivation Participative approach Partners Communication
The**G**ender**E**quality**Tool** for Generation**Z**	Gender Equality	Online training	Research and practice-based evidence	Create a supportive environment for health Develop personal skills Re-orient health services	Resources Experience Mobilization Motivation Partners Communication
How to select mental health program providers for sports clubs	Mental health program provision	Leaflet	Research based evidence	Strengthen community action for health	Mobilization Partners
Integration of Refugees through Sports	Refugee integration through sports	Online training	Research based evidence	Create a supportive environment for health Strengthen community action for health Develop personal skills Re-orient health services	Education Mobilization Participative approach Partners
IOC Mental Health in elite athletes tool	Mental health	Leaflet	Research and practice-based evidence	Create a supportive environment for health Develop personal skills Re-orient health services	Education Dynamic Motivation Partners
Keep youngster involved	Youth sports drop out	Game	Research and practice-based evidence	Create a supportive environment for health Strengthen community action for health	Mobilization Motivation Participative approach Communication
Sports and physical activity for people with mental health problems: a tool for the sports sector	Mental health	Leaflet	Practice-based evidence	Build healthy public policy Create a supportive environment for health Strengthen community action for health Develop personal skills	Planning Resources Goals Mobilization Partners Communication
STOP sports injuries	Injury prevention	Website	Research and practice-based evidence	Create a supportive environment for health Strengthen community action for health Develop personal skills	Education Resources Dynamic Experience Feasibility Goals Mobilization Monitoring Motivation Participative approach Partners Communication
Sports Clubs for Health	Physical Activity participation	Leaflet and online training	Research and practice-based evidence	Build healthy public policy Create a supportive environment for health Develop personal skills	Planning Feasibility Goals Motivation Communication
SUGAPAS	Health behavior	Online training	Research and practice-based evidence	Develop personal skills	Education Resources
Supporting mental wellbeing in community sports	Mental health	Leaflet, video and checklist	Research based evidence	Create a supportive environment for health Develop personal skills	Education Dynamic Feasibility Mobilization Participative approach Partners Communication

### Step 5: finalization of the HPSF guidelines

The final guidelines includes 151 pages, structured across four chapters: analyses and monitors HP in a NSF, is inspired by case studies and examples of NSF investment in HP, implements the HPSC framework, identifies tools to support HPSF and HPSC. The guidelines can be approached in different ways; however, it is recommended to proceed by reading the first chapter and application of the evaluation criteria and acknowledgement of the HPSF stage for the NSF. Then the case studies, intervention components and tools can be used to move on to the HP development in a sports federation, as each of them are linked to a specific strategy of the HPSC framework.

## Discussion

The HPSF guidelines have been designed to clarify the theoretical concept of a HPSF and support its implementation through case studies, intervention components and tools. The guidelines are designed to act as a springboard for NSF to acknowledge, reinforce and further foster their investment in HP. This work is aligned with recent research efforts to clarify the theoretical tenets of a HPSC, as a previous literature review has shown that the application of the settings-based approach to HP has to be tailored to the context of the setting and core business (40) of the organization. For example, schools, universities and even cities have different characteristics compared to sports clubs (41), which are typically run by volunteers, have sports provision as their main aim, do not always own their facilities (13) and depend on NSF regulations, support and guidance (42). Moreover, a recent literature review showed a poor application of the settings-based approach in sports clubs, with programs principally targeting sports participants and lacking support for clubs to make organizational changes (8). The HPSF Guidelines consider the four determinants of health: organizational, social, environmental and economic, and go beyond traditional education program for coaches or health information for sports participants (43), supporting the implementation of more complex HP programs.

Another important aspect of these guidelines is that they constitute the first offer of theory-based intervention components to implement within NSFs, whereas previous studies have principally used observational studies or did not root their intervention in a theoretical framework. Nevertheless, no empirical data have been collected scientifically on the application of these intervention components, and there is a need to test their application and outcomes. In that regard, the creation of HPSF indicators, considering the whole system of a NSF, highlight key outcomes of the development of HP by NSF, therefore providing a first attempt for a self-evaluation tool for NSF. In line with previous publications on the HPSC model (44), evaluation indicators can help to clarify the resources, activities, outcomes and impact of HP development by NSF, and could help government authorities, and local sports clubs to clearly call on their fulfillment by NSFs.

The analysis of existing tools showed that most of them are centered on a single dimension of health (mental, social, physical, spiritual and community), that they are often on a single health topic and that they do not cover HP as a process, more as a short-term program, that in turn, does not support sustained improvement to the health of sports club members. These findings are similar to results found in sports clubs, e.g., in Australia only 3% of the sports clubs had a policy on multiple health topics (45). In France, a case study on 8 exemplary HP projects has demonstrated that each project targeted a single health topic (24). Interestingly, creating a supportive health environment was the most targeted strategy of the Ottawa Charter (39), where reorienting health services was the least implemented. There is a need to move from interventions directly targeting sports participants towards a whole system approach (46). In regard to creating public health policy, efforts are still needed in sport on policy development, having been acknowledged in research as the biggest weakness of sports clubs (47). In addition, creating a supportive environment is the Ottawa Charter strategy which is the most aligned to sports clubs most developed HPSC strategies, like mobilization, resources, partners and education, which are also linked to identifying financial and human resource support to develop HP. This is in line with a request from sports clubs, identified in a previous concept mapping study among French sports clubs (19). A more challenging result concerns the few tools mentioning the experience and the monitoring of implementation strategies. The evaluation of HP in sports clubs is not undertaken nor encouraged in these tools, whereas previous work has shown that HP project managers in sports clubs lacked tools and methods to evaluate their actions (24) and a previous literature review has demonstrated the paucity of use of validated measurement to evaluate the application of the HPSC approach (8). As the settings-based approach is challenging to evaluate (48), robust methods to investigate the (cost) effectiveness of policy and practice at multiple levels, using multiple strategies have already been used with health promotion interventions in sports clubs (26, 27).

The developed guidelines will be published on the World Health Organization’s website. NSFs can use these guidelines to establish appropriate governance to support HP and embed it in existing policies and practice, identify tools and examples to support affiliated sports clubs to invest in HP, develop programs supporting HP in and through sports and provide evidence that the NSF is playing an enhanced role in society.

### Strengths and limitations

The rigorous method and multiple steps used to create the HPSF Guidelines helped to produce a theory based and evidence informed tool, offering the opportunity to develop multi-levels and multi-strategies, in accordance with public health researchers’ recommendations (49). Moreover, the final guidelines have been reviewed by 3 practitioners beyond the group, to facilitate a transfer into practice.

Nevertheless, several limitations to the present study should be noted. First, no new literature review on how NSF undertake HP was conducted to evaluate the current state of the evidence, as well as barriers and facilitators to its implementation. Second, the present guidelines is created in English, limiting its use and dissemination in some countries and most of the researchers involved came from Europe, possibly limiting its application to Asia, Africa or North America. Third, NSFs were only involved in the final proof reading (50), as no co-construction process was undertaken through participatory research.

## Conclusion

The article provides theoretical foundations, analysis of practical tools and strategies to support NSF investment in HP. It contributes to the development of HP in organized sports by providing a definition of HPSF, illustrating the application of the setting-based approach to HP to NSFs and providing intervention components linked with strategies in the health promoting sports club’s intervention framework. The intervention components, guidelines and tools provide practitioners, including NSFs, clear and useful guidance on how to promote health. Future research needs to further evaluate the feasibility and acceptability of this theory based guidelines, their effectiveness in delivering health outcomes as well as their adaptability to different cultural contexts.

## Data availability statement

The original contributions presented in the study are included in the article/supplementary material, further inquiries can be directed to the corresponding author.

## Ethics statement

Ethical review and approval was not required for the study on human participants in accordance with the local legislation and institutional requirements. The patients/participants provided their written informed consent to participate in this study.

## Author contributions

AH, SG, AV, and MW have contributed to research design and method definition. All others authors have contributed to shared knowledge, toolkit evaluation, reading of successive version of the guidelines. AH, AV, and CW have secured the funding to generate these guidelines. All authors contributed to the article and approved the submitted version.

## Funding

This project has received funding from the European Union’s Horizon 2020 Research and Innovation Programme under the Marie Skłodowska-Curie grant agreement No. 101028401. This work was funded by a grant from the World Health Organization in partnership with Santé publique France, Université de Lorraine and Université Côte d’Azur.

## Conflict of interest

The authors declare that the research was conducted in the absence of any commercial or financial relationships that could be construed as a potential conflict of interest.

## Publisher’s note

All claims expressed in this article are solely those of the authors and do not necessarily represent those of their affiliated organizations, or those of the publisher, the editors and the reviewers. Any product that may be evaluated in this article, or claim that may be made by its manufacturer, is not guaranteed or endorsed by the publisher.

## Footnotes

^1^ https://erasmus-plus.ec.europa.eu/projects/search

^2^ https://www.insee.fr/fr/metadonnees/definition/c1258
